# The Safety of Slaughterhouse Workers during the Pandemic Crisis

**DOI:** 10.3390/ijerph18052633

**Published:** 2021-03-05

**Authors:** Claudiu Ștefan Ursachi, Florentina-Daniela Munteanu, Gabriela Cioca

**Affiliations:** 1 Faculty of Food Engineering, Tourism and Environmental Protection, “Aurel Vlaicu” University of Arad, 2-4 E. Drăgoi Str., 310330 Arad, Romania; claudiu.ursachi@uav.ro; 2 Preclinical Department, Faculty of Medicine, Lucian Blaga University of Sibiu, 550024 Sibiu, Romania; gabriela.cioca@ulbsibiu.ro

**Keywords:** slaughterhouse, workers’ safety, pandemic, SARS-CoV-2

## Abstract

The working conditions in a slaughterhouse are difficult because of the low temperatures, high humidity, and little natural light. Therefore, in these facilities, there is a high demand in the maintenance of strict hygiene rules. Lately, the new SARS-CoV-2 pandemic situation has brought new challenges in the meat industry, as this sector has to maintain its operability to supply the meat and meat products demanded by the consumers. In this challenging period, the safety of the workers is as important as keeping the high demands for the safety of the meat and meat products along with consumer confidence. This paper aims to give an overview of the risks associated with the SARS-CoV-2 virus transmission between the workers in slaughterhouses and to evaluate the stability and infectivity in the working environment of these facilities. Considering the persistence of this virus on different surfaces and the environmental conditions affecting its stability (temperature, relative humidity, and natural light), in the study we proposed several short-, medium-, and long-term preventive measures for minimizing the potential threats of the actual pandemic.

## 1. Introduction

Meat and meat product consumption has registered an increasing trend since the early 1960s [1]. Basu recently discussed this tendency [2], and showed that the supply of meat products increased by 204% in the period of 1960–2010, while Katare [3] reported a 500% increase in meat consumption in the period 1992–2016. 

Even though the consumption of proteins from plant-based products has been maintained stably in the last 60 years, meat and meat products are the major source of proteins, minerals, and vitamins [4], and represent about 30% of the total calorie consumption in the European Union [5]. Besides the risks associated with human health, the negative impact on the environment and animal welfare should also be considered [3,4,5]. 

Nowadays, the meat industry in the European Union comprises over 32,000 companies with over one million employees. This industry represents 1.53% of the gross domestic product (GDP) [6].

Such a high demand for meat and meat products leads to intense activity in slaughterhouses around the world. The type of slaughtered animal differs between countries as a consequence of wealth and culture [7].

Generally, because of the different services that are taking place in the slaughterhouses, there is a continuous need for facilities and equipment that assure a safe production of meat and meat products designed for human consumption, along with the safety of the workers. 

In general, a slaughterhouse can encounter all types of contaminants, physical, chemical, and biological. The growth of pathogens is influenced by how the animals are slaughtered, eviscerated, and stored, and therefore, the potential of contamination of the meat and meat production is high [8]. The presence of bioaerosols in the working environment can lead among the exposed workers to respiratory disorders, chronic inflammatory diseases of the respiratory system, acute toxic affections, infectious diseases, and occupational asthma [9].

Meanwhile, because of the continuous contact of the workers with biological materials, they can be exposed to zoonotic pathogens [10], a fact that makes the abattoirs to be considered major sources of occupational exposure. 

By the end of 2019, a COVID-19 outbreak emerged in Wuhan, China, and the World Health Organization (WHO) declared it a pandemic in February 2020. Some authors have considered that, because of the dynamics of transmission of the SARS-CoV-2 virus, this should be assimilated to a zoonotic illness [11,12].

Considering the congestion of the workers in the slaughterhouses, any possible zoonotic outbreak will be worsened, and the application of the existing bio-risk mitigation measures could be a challenge.

This paper focuses on the evaluation of the risks associated with potential infection with COVID-19 of slaughterhouse workers. The study considers the working environment and most used inanimate surfaces during the activities in these facilities. Moreover, some measures for the safety of the workers are proposed.

## 2. Materials and Methods

For the present paper, we carried out a critical literature review, and the most relevant publications were retrieved from different journal databases. 

The search of the literature focused on papers published in ScienceDirect, SpringerLink, and ProQuest Central databases. The authors considered articles published from the beginning to the end of 2020, including the available accepted journal papers planned for volumes with appearance in 2021. The following keywords were selected and used with Boolean operators: slaughterhouse, SARS-COV-2, COVID-19, zoonosis, worker safety, risks, inanimate surfaces, persistence, and environmental conditions. The present study excluded, although consulted, the manuscripts present in the preprints server that are figured as preliminary reports and have not been peer-reviewed. The investigators evaluated the screened material and considered the most appropriate information regarding the workers’ safety in slaughterhouses. Considering the structure of the present paper, the number of consulted references is assigned as follows: (i) 13 references for zoonotic pathogens; (ii) 25 papers for the stability of the SARS-CoV-2 virus in the environment; (iii) 14 references for the environmental factors that favor the transmission of pathogens in slaughterhouses; and (iv) 11 articles for the proposed safety measures.

## 3. Zoonotic Pathogens

Zoonosis is referred to as infectious diseases that are provoked by a pathogen that is transmitted from an animal to a human. Zoonotic pathogens can comprise bacteria, viruses, or parasites that can be spread to humans through food products, water, or the environment. Some of the zoonotic viruses that can be transmitted to slaughterhouse workers refer to viruses that provoke diseases, such as contagious ecthyma, influenza, rotavirus, Newcastle disease, cowpox, and Louping ill [13]. These viruses can be transmitted mainly through direct contact with the contaminated material [7,8,10,12,13,14]. 

Besides the viruses mentioned above, there is also the class of *Coronaviridae* that is further divided into four genera: alphacoronavirus, betacoronavirus, gammacoronavirus, and deltacoronavirus, however, it was found that only alpha- and betacoronaviruses are infectious for humans [15,16]. Coronaviruses are endemic, and most of the strains cause common colds, but some of the strains provoke severe lower respiratory tract infections, and might be lethal [17,18,19,20]. 

Up until now, the most known outbreak provoked by the coronaviruses was in China in 2002, the severe acute respiratory syndrome coronavirus (SARS-CoV), and this spread to 33 countries [21]; then, in 2012 in the Arabian Peninsula, there was the Middle East respiratory syndrome coronavirus (MERS-CoV) [22].

Since the end of 2019, a new strain of the severe acute respiratory coronavirus, SARS-CoV-2, also named COVID-19, became a health emergency that is of international concern [23]. The COVID-19 virus is very contagious and extremely infectious compared to SARS-CoV and MERS-CoV [21,22,24].

The novel coronavirus, named severe acute respiratory syndrome coronavirus 2 (SARS-CoV-2), belongs to the genus Betacoronavirus, and is an RNA virus [25,26,27,28]. Some authors have shown that the COVID-19 virus is of bat origin, and that the detailed analysis of the genome of SARS-CoV-2 concluded that 380 amino acids are substituted, a fact that contributes to the pathogenic divergence of this new virus [28,29,30,31,32].

The transmittance of the virus can be through direct contact between the infected patient and a healthy person, through sneeze or cough respiratory droplets, and from contaminated surfaces or environments [24,33]. 

The safety of the workers of slaughterhouses is always a challenge, but nowadays, because of the new COVID-19 pandemic situation, this needs special attention because of the environmental working conditions. These working conditions refer mainly to the high humidity associated with the amount of water used for washing the carcasses, equipment, and surfaces, low temperatures, and the presence of residual organic material [7,8,9,34].

Another issue that should be considered is that, working at temperatures between 4 and 10 °C, the breath of the workers will produce condensation of the exhaled air and rapid humidification of the protective masks, a fact that will contribute to an inappropriate filtration of the air or even to inappropriate wearing of the protection equipment.

## 4. Stability of the SARS-CoV-2 Virus in the Environment

**Figure 1:**
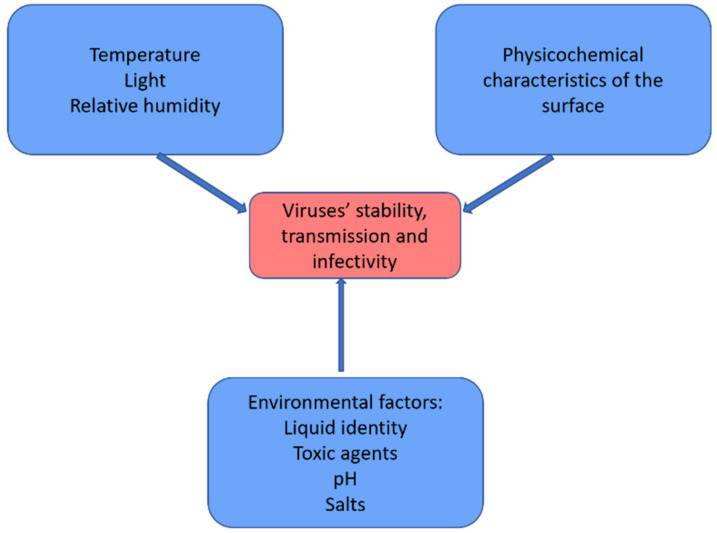
Environmental conditions affecting the stability of the viruses.

The attachment of the virus on inanimate surfaces is dependent on the physicochemical properties of the surface [35,36,37]. Vasickova et al. showed that the adsorbed amount of viruses on surfaces plays an important role in the viruses’ survival and their infectivity on mammalians [38], even though other studies demonstrated that strong irreversible adsorption on surfaces might lead to inactivation of viruses [36]. On the other hand, environmental conditions play an important role in the stability and infectivity of the viruses [39,40,41], as shown in Figure 1. 

Recently, several papers have been published containing information about the persistence of coronaviruses, and the researchers concluded that human coronaviruses could persist on different surfaces for up to nine days, but, fortunately, a thorough disinfection procedure would lead to virus inactivation [39,40,42,43,44,45,46,47,48,49,50,51,52].

Van Doremalen et al. [50] showed that the SARS-CoV-2 virus is more stable on plastic and stainless steel (72 h) than on cardboard (24 h) and copper (4 h), while Ren et al. [53] concluded that most viruses are persistent on the surface for a few days, noting that the absorbent materials are safer than other materials. The results obtained by Chin et al. [49] for the stability of SARS-CoV-2 on different surfaces indicated similar values to those reported by van Doremalen, while the study of virus stability at different temperatures revealed a decay of the viral load with the increase in temperature. Aboubakr et al. [39] published an interesting outcome and concluded that the persistence of the SARS-CoV-2 virus is significantly reduced on copper. 

The studies of the influence of temperature and relative humidity on the stability of the COVID-19 virus on non-porous surfaces indicated a rapid decay of the viral load if either temperature or humidity increased [54]. Moreover, the persistence of the virus is higher when the temperature is lower [42,43,46,54].

The persistence of the COVID-19 virus in aerosols is another aspect that should be considered. While in one study [50] it was reported that the virus was still detectable after 3 h, in another study, it was reported that the virus retained its infectivity for more than 16 h [44].

Special attention should also be paid to the presence of the SARS-CoV-2 virus in wastewaters, as the virus was also detected in feces and urine [55,56,57]. By knowing this aspect, it is commendable to monitor the load of the virus in wastewater from slaughterhouses, as this can be an indicator for future planning of the mitigation of risks. 

## 5. Environmental Factors That Favor Transmission of Pathogens in Slaughterhouses

Like all of the other cold food processing establishments, slaughterhouses and meat processing factories are considered as vectors for the spread of COVID-19. According to the literature, the major causes that are contributing to a higher risk of virus transmission in the meat sector are the microclimates in the abattoirs, materials used for the equipment inside of the slaughterhouses, the type of ventilation of the air, organization of the workstations, the type of equipment used, the ability of the workers to respect the safety rules, the awareness of the workers in respecting the prevention measures in case of infection with the COVID-19 virus, the assurance of transportation in safe conditions, and respecting the minimum recommended distance between individuals.

Meat plants’ microclimate (low temperature, high relative humidity, absence of sunlight) are favorable for coronavirus to thrive and spread [39]. Experiments have shown that SARS-CoV-2 has high stability at 4 °C, with a reduction in infectious titer of only around 0.7 log_10_ after 14 days. On the other hand, when the incubation temperature was increased to 70 °C, the inactivation times were reduced to only 5 min [49]. In another study, Wang et al. [58] evaluated the stability of SARS-CoV-2 in different conditions. They reported that the virus was stable at least 24 h at 37 °C, but it was inactivated by heating at 56 °C for 30 min. Other experiments showed that the virus could survive on non-porous surfaces for up to 28 days at 20 °C and 50% relative humidity [48], while others reported that the infectious SARS-CoV-2 could survive at least 14 days at 4 °C in domestic and hospital wastewater and de-chlorinated tap water [59].

Metallic and plastic surfaces maintain live viruses for a longer time than other materials. At an ambient temperature (22 °C) and 65% relative humidity, no infectious virus could be detected on stainless steel after seven days, on glass after five days, on wood and textiles after two days, and on tissue paper after 3 h [49]. According to van Doremalen et al., the survival of coronaviruses on metal surfaces also varies with the type of metal. The survival period is longer on zinc and stainless steel and shorter on nickel, brass, and copper. For example, the estimated median half-life of SARS-CoV-2 was around 5.6 h on stainless steel [50].

Air conditioning systems in slaughterhouses may spread virus particles and therefore increase the risk of infection. The information about the persistence of SARS-CoV-2 in the air is known only from hospital environments. Air sample analysis from a COVID-19 ward at a hospital in Wuhan, China indicated the presence of virus RNA in the air even at 4 m from patients [60]. In another study, it was shown that SARS-CoV-2 persists in aerosols for 3 h, indicating that aerosol transmission could be possible, since the virus remains viable [50]. Fears et al. [44] also confirmed in their study that aerosolized SARS-CoV-2 continued to be infectious even after 16 h at room temperature.

It is well-known that the equipment in slaughterhouses is noisy, i.e. grinders, mixers, tumblers, bone saws, and crushers, and these require workers to talk loudly, increasing the risk of spreading virus-infected droplets. Moreover, the specific working conditions in proximity to one another, with increasing line speeds and the internal circulation in these plants, make it difficult to keep a safe distance between the workers, and certainly contributes to the spread of the virus. 

In most European countries, the meat industry depends on migrant and cross-border workers. Because of the need to save as much as possible from their salaries, these workers share rented dormitories with several other people, and most of the time, these facilities do not meet the minimal hygiene standards. Therefore, respecting social distancing in these accommodations is almost impossible. Moreover, the workers are transported together on buses, without ensuring social distancing during transport. 

Another aspect that is contributing to the virus spreading between workers is that, in the case of COVID-19 symptoms, most of them do not report their health status and continue to go to work because of the fear of losing their job.

In meat factories, cleaning is performed by high-pressure washing of floors, surfaces, and equipment. These methods produce large amounts of droplets and generate aerosols. If the floor and surfaces are contaminated by infected workers, viruses will be spread into the air, and could be a potential risk of infection for other workers [61].

Another problem frequently met in slaughterhouses with a big number of foreign employees is the difficulty of implementing biosecurity mitigation measures that can be respected by a multicultural working community that might not speak and/or understand the native language spoken in the host country.

The preventive health and safety measures adopted to protect workers are also affected by the decreased frequency of labor inspections during the pandemic period.

A rigorous mitigation action plan is necessary, as the vulnerability to natural, biological, chemical, or any other types of hazards might not be prevented through the existing public health risk mitigation measures [62]. 

## 6. Proposed Preventive Measures in Slaughterhouses during the Pandemic

According to some public health authorities, like the Centers for Disease Control and Prevention (CDC), World Health Organization (WHO), and Food and Drug Administration (FDA), there is no evidence for severe acute respiratory syndrome coronavirus (SARS-CoV-2) direct contamination or transmission by the ingestion of contaminated food [63,64]. 

However, it cannot be ignored that there is a possibility of spreading the novel coronavirus through contaminated food or food packages, as there are insufficient and edifying studies reporting on this subject [65]. Furthermore, a wide variety of viruses, like Hepatitis E, Adenovirus, Enterovirus, Astrovirus, and Coronavirus (including SARS-CoV-1 and influenza), could contaminate foods, and are associated with foodborne transmission [66,67].

Recently, some authors have reported on foods and food packaging materials contaminated with SARS-CoV-2. Harbourt and collaborators reported that SARS-CoV-2 remained largely viable on pork skin after 14 days at 4 °C and four days at 22 °C with a relative humidity of 40–50% [68]. Because meat processing, packing procedures, and storage are performed in general between 4–8 °C, it is possible that viral shedding from contaminated workers, in the absence of adequate protective equipment, would remain viable for a long period on meat and meat product surfaces.

In another study, Fisher et al. demonstrated in their laboratory experiment the survival ability of SARS-CoV-2 at temperatures and time associated with refrigerated (4 °C) and frozen food (−20 °C) storage and transportation conditions. On pork, salmon, and chicken samples inoculated with SARS-CoV-2, the viruses maintained their infectivity even after 21 days for both refrigerated and frozen samples [69].

In June 2020, in one of the Beijing agricultural product markets, SARS-CoV-2 was identified on a cutting board used for processing imported salmon. Not much longer after that, in July 2020, the coronavirus was detected on the packaging materials of frozen shrimp imported from Ecuador. In August, local authorities of Guangdong confirmed the presence of SARS-CoV-2 on frozen chicken surfaces imported from Brazil [64]. 

The detection of SARS-CoV-2 on frozen foods suggests that these cases are not random, isolated incidents, but rather are alarming signs that viral contamination and foodborne transmission may present a systematic risk in the ongoing pandemic. 

Although there is no certainty of food transmission in slaughterhouses and meat factories, due to the large number of infestations reported among workers in this sector of the food industry, it is necessary to take safety measures regarding food contamination from infected people. Food handlers infected with COVID-19 can expel droplets by breathing, talking, coughing, and sneezing, contaminating the foods or the packaging materials in proximity [70]. Additional safety measures are especially required for refrigerated and frozen products due to the high ability of the virus to survive under these conditions. The continuous low temperature and high humidity environments during the entire food processing chain of cold-storage foodstuffs create a favorable condition that can extend the survival of SARS-CoV-2. 

The meat processing sector, as well as the entire food industry, needs to prepare for epidemics and pandemics with new technological solutions, including a greater degree of automation and robotics, to reduce human food contact and the human density in workplaces to minimize food waste and ensure food safety [71].

The plan for risk minimization of COVID-19 in the meat industry should consider two directions, (i) in terms of food safety for the prevention of contamination of susceptible food or packaging materials and (ii) concerning the employees’ safety to ensure safe working conditions [72].

For the safety of the workers in slaughterhouses, we are proposing short-, medium-, and long-term measures. Regarding the short-term, special attention should be paid to the (i) identification and isolation of contaminated persons; (ii) periodic training of workers on virus spreading, the correct methods of prevention and sanitation, and the importance of wearing protective equipment; (iii) active symptoms screening, including temperature monitoring of all workers and visitors; (iv) using adequate personal protective equipment (PPE); (v) strict observance of effective hygiene practices; (vi) maintaining physical distancing both on the production line and during breaks; and (vii) the use of appropriate and effective disinfectants.

The medium- and long-term measures should focus on (i) fast and efficient methods of identifying contaminated persons or areas; (ii) increasing the degree of automation and robotization of technological processes; (iii) finding effective sanitation alternatives that do not affect the health of workers and/or pollute the environment; and (iv) finding technological solutions, such as biosensors or nanosensors, to identify the virus on equipment, workspaces, and surfaces of the food or food packaging.

## 7. Conclusions

Based on the findings already published in the literature, slaughterhouse managers should consider more rigorous preventive measures implemented in their safety management systems for combating the SARS-CoV-2 virus, and a good start could be from the measures proposed in Section 6. Therefore, the meat processing companies should consider an immediate evaluation of the risks in the actual pandemic crisis and implement hierarchical measures that prevent such outbreaks. These should include, besides all of the measures that reduce the crowding of the workers, different hours for breaks and installing barriers between workstations. Moreover, for facilitated communication between the workers, special devices and systems should be provided for preventing the spread of the virus. 

Special attention should be paid to the workers’ awareness and hygiene, along with a thorough check of their health status to maintain the safety of all workers. An important aspect is to apply a good communication procedure to assure that all of the workers have understood the application of the safety measures and that the language hindrances are overcome. Not of less importance is the necessity to use more frequently the disinfectants appropriate for use in the meat industry, and disinfect more often, especially surfaces that are proven to maintain the viability of the COVID-19 virus for a longer time.

Last but not least, special regulations should be adopted for the encouragement of the workers to declare any symptoms of illness and self-isolate. This should be sustained by an adequate payment that will dispel the fear of unemployment.

## Data Availability

Data is contained within the article.
